# An Antiherpesviral Host-Directed Strategy Based on CDK7 Covalently Binding Drugs: Target-Selective, Picomolar-Dose, Cross-Virus Reactivity

**DOI:** 10.3390/pharmaceutics16020158

**Published:** 2024-01-23

**Authors:** DongHoon Yu, Sabrina Wagner, Martin Schütz, Yeejin Jeon, Mooyoung Seo, Jaeseung Kim, Nadine Brückner, Jintawee Kicuntod, Julia Tillmanns, Christina Wangen, Friedrich Hahn, Benedikt B. Kaufer, Frank Neipel, Jan Eickhoff, Bert Klebl, Kiyean Nam, Manfred Marschall

**Affiliations:** 1 Qurient Co., Ltd., C-Dong, 242 Pangyo-ro, C801 Bundang-gu, Seongnam-si 13487, Republic of Korea; 2 Institute for Clinical and Molecular Virolosgy, Friedrich-Alexander-Universität Erlangen-Nürnberg (FAU), Schlossgarten 4, 91054 Erlangen, Germany; 3 Institute of Virology, Freie Universität Berlin, Robert-von-Ostertag-Straße 7–13, 14163 Berlin, Germany; 4 Lead Discovery Center GmbH, Otto-Hahn-Straße 15, 44227 Dortmund, Germany; 5 The Norwegian College of Fishery Science UiT, Arctic University of Norway, 9037 Tromsø, Norway

**Keywords:** cytomegalovirus infection, major human pathogen, virus-supportive host kinases, cyclin-dependent kinase 7 (CDK7), covalently CDK7-binding warheads, distinct mode of drug targeting, picomolar effective concentrations, drug synergism with maribavir, novel antiherpesviral strategy

## Abstract

The repertoire of currently available antiviral drugs spans therapeutic applications against a number of important human pathogens distributed worldwide. These include cases of the pandemic severe acute respiratory coronavirus type 2 (SARS-CoV-2 or COVID-19), human immunodeficiency virus type 1 (HIV-1 or AIDS), and the pregnancy- and posttransplant-relevant human cytomegalovirus (HCMV). In almost all cases, approved therapies are based on direct-acting antivirals (DAAs), but their benefit, particularly in long-term applications, is often limited by the induction of viral drug resistance or side effects. These issues might be addressed by the additional use of host-directed antivirals (HDAs). As a strong input from long-term experiences with cancer therapies, host protein kinases may serve as HDA targets of mechanistically new antiviral drugs. The study demonstrates such a novel antiviral strategy by targeting the major virus-supportive host kinase CDK7. Importantly, this strategy focuses on highly selective, 3D structure-derived CDK7 inhibitors carrying a warhead moiety that mediates covalent target binding. In summary, the main experimental findings of this study are as follows: (1) the in vitro verification of CDK7 inhibition and selectivity that confirms the warhead covalent-binding principle (by CDK-specific kinase assays), (2) the highly pronounced antiviral efficacies of the hit compounds (in cultured cell-based infection models) with half-maximal effective concentrations that reach down to picomolar levels, (3) a particularly strong potency of compounds against strains and reporter-expressing recombinants of HCMV (using infection assays in primary human fibroblasts), (4) additional activity against further herpesviruses such as animal CMVs and VZV, (5) unique mechanistic properties that include an immediate block of HCMV replication directed early (determined by Western blot detection of viral marker proteins), (6) a substantial drug synergism in combination with MBV (measured by a Loewe additivity fixed-dose assay), and (7) a strong sensitivity of clinically relevant HCMV mutants carrying MBV or ganciclovir resistance markers. Combined, the data highlight the huge developmental potential of this host-directed antiviral targeting concept utilizing covalently binding CDK7 inhibitors.

## 1. Introduction

Considering the current repertoire of approved antiviral drugs, it appears striking that the vast majority is provided by direct-acting antivirals (DAAs). This may have practical reasons in the history of antiviral drug development, since the strategy was originally derived from the early recognition of virus-encoded targets and target structures that are unique or differ from their host orthologs. DAAs have been proven to be clinically highly expedient, especially in antiviral therapy and prevention of infections with human retro-, herpes-, influenza, and hepatitis viruses. The achievements and benefits of DAA treatment, however, can be strongly limited, in particular with long-term therapeutic or prophylactic treatments, the induction of viral drug resistance, or unwarranted side effects. For these reasons, we addressed the question of alternative or additional use of host-directed antivirals (HDAs). Host-directed drugs, in particular when recognizing pathogenesis-related mutants of target proteins, have been greatly successful in areas spanning the treatments of tumor, metabolic, infectious, and inflammatory diseases [1,2,3,4,5,6,7,8,9,10,11,12,13,14]. However, within the field of antiviral therapy, this topic misses in-detail experience, since only quite few HDAs have been clinically approved and utilized in therapeutic settings thus far.

Thus, concerning antiviral HDA development, ribavirin has been the first and only example of an antiviral HDA for a long time that exerts broad-spectrum antiviral activity. It is based on a complex mode of action (MoA) which includes the inhibition of host inosine monophosphate dehydrogenase (IMPDH), subsequently leading to depletion of the intracellular GTP pool [15,16,17,18,19,20]. Maraviroc, a human chemokine receptor CCR5-specific coreceptor antagonist, provides activity against human immunodeficiency virus type 1 (HIV-1). The drug selectively binds to the host CCR5 present on the membrane of CD4-positive T cells, preventing the interaction of HIV-1 gp120 and CCR5 necessary for CCR5-tropic HIV-1 to enter cells [21,22,23,24]. Quite recently, bulevirtide was approved as another HDA that comprises receptor-blocking entry activity and shows antiviral potency against hepatitis D and B viruses [25,26,27,28,29,30].

Of specific importance, maribavir (MBV) was approved by the U.S. FDA in 2021 as the first kinase inhibitor within the entire field of antiviral therapy. However, due to the fact that MBV is directed to and inhibits the HCMV-encoded cyclin-dependent kinase ortholog vCDKpUL97, thus exerting a selective pressure on the variable sequence markers of its kinase domain, the issue of drug resistance formation could not be solved yet. Thus, on the basis of this kinase-directed strategy, our goal was to develop a first host-directed mono-targeted enzymatic inhibitor (i.e., a CDK7 inhibitor with optimized antiviral properties). As a specific advantage of this strategy, the regulatory functions of CDK–cyclin complexes appear to be redundant within the host cell physiology [31,32,33,34,35,36,37,38,39,40,41] but can be highly restrictive for viral replication and the pathogenesis-determining viral load. In particular, concerning the role of CDK7, it has previously been demonstrated that a functional depletion of CDK7 can be cross-compensated by related CDK-cyclin complexes [31] and is even tolerated in the non-embryonal adult tissues of knock-out mice [42]. On the other hand, CDK7 activity proved to be strictly required for the high efficiency of viral replication [41,43,44,45,46,47,48,49,50]. Interestingly, although the depletion of CDK7 function may lead to drastic cytotoxic effects and apoptosis in melanoma cells [51], this was not the case in non-tumor cells or herpesvirus-infected cells or mice [52]. Based on these findings, we built on the experimental approach to target the major virus-supportive function of host kinase CDK7, especially through the development of 3D structure-derived covalently binding warhead compounds. Here, our concept is demonstrated in terms of the three compounds QRS6, QRS7, and QRS9, with all tracing back to the parental compound LDC4297, and the putative future prospects of preclinical and clinical antiviral drug development are discussed.

## 2. Materials and Methods

### 2.1. Antiviral Compounds

The synthesis and antiviral characterization of the parental, selective CDK7 inhibitor LDC4297 (Lead Discovery Center GmbH, Dortmund, Germany) have previously been described in detail [43,44,45,47,52,53,54]. The covalently binding derivatives QRS6, QRS7, and QRS were designed and synthesized by Qurient Co., Ltd. (C-dong, Pangyo-ro, Bundang-gu, Seongnam-si, Gyeonggi-do, Republic of Korea; Figure S1). The antiherpesviral drugs ganciclovir (GCV; Sigma-Aldrich, St. Louis, MO, USA), cidofovir (CDV; Pharmacia & Upjohn S.A., Luxembourg, Luxembourg), and maribavir (MBV; MedChemExpress, Monmouth Junction, NJ, USA) were purchased from the indicated sources and used as reference compounds. Stock aliquots were prepared in DMSO (Sigma-Aldrich, USA) at 10 mM and stored at −20 °C.

### 2.2. Cultured Cells and Cytomegalovirus Infection

Primary human foreskin fibroblasts (HFFs, C0045C, Thermo Fisher Scientific, Waltham, MA, USA) were cultured in MEM (21090055, Thermo Fisher Scientific) supplemented with 10% FCS, 1× GlutaMAXTM (35050038, Thermo Fisher Scientific), and 10 g/mL gentamicin (22185.03, SERVA, Heidelberg, Germany) at 37 °C, 5% CO_2_, and 80% humidity. Primary murine and guinea pig fibroblasts as well as iSLK.219 cells were cultured in MEM (21090055, Thermo Fisher Scientific) under the conditions described for HFFs. Lymphoid J-Jhan T cells and Akata-BX1-GFP B cells were cultured in RPMI-1640 medium (Thermo Fisher Scientific, USA) supplemented with 10% FCS, 1× GlutaMAXTM, and 10 g/mL gentamicin. HCMV strain AD169 and its green fluorescent protein (GFP)-expressing recombinant AD169-GFP were used for infection experiments at a multiplicity of infection (MOI) of 0.25 (i.e., 0.25 GFP-FU/mL at 7 d p.i., equating to an initial MOI of 0.01–0.001 IE-FU/mL at the time point of infection), as recently determined to be optimal for this GFP-based antiviral assay (thus resulting in a maximal coverage of 25% virus-positive cells after 2.5 rounds of the 3 d replication cycle of HCMV). After inoculum–cell adsorption during incubation at 37 °C for 90 min, the viral inocula were then replaced by fresh growth medium, optionally containing the adjusted concentrations of antiviral compounds.

### 2.3. Human and Animal Herpesviruses and Drug-Resistant Cytomegalovirus Mutants

For the infection experiments, stocks of the following recombinant, reporter protein-expressing viruses (GFP or YFP (i.e., green or yellow fluorescent protein, respectively)) were propagated (in permissive cells), determined in their infectious titers performing the respective reporter assays, stored by aliquots at −80 °C, and used for the assessment of antiviral drug activities: human cytomegalovirus HCMV AD169-GFP (HFFs) [55]; murine cytomegalovirus MCMV C3X-GFP (MEFs) [56]; guinea pig cytomegalovirus GPCMV v403-GFP (GPEFs) [43]; human herpesvirus type 6A HHV-6A U1102-GFP (J-Jhan) [57]; herpes simplex virus type 1 HSV-1 166v VP22-GFP (HFFs) [43]; varicella zoster virus VZV Oka-GFP (HFFs) [58]; Epstein-Barr virus EBV Akata-GFP (Akata-BX1-GFP carrier cells; kindly provided by Lindsey Hutt-Fletcher and Rona Scott, Dept. Microbiol. Immunol., LSUHSC Univ., Shreveport, LA, USA) [59,60]; and Kaposi’s sarcoma-associated herpesvirus KSHV rKSHV.219-GFP/RFP (in iSLK.219 carrier cells) [61]. The genetic generation, propagation, and use of reporter protein-expressing drug resistant mutants of HCMV TB40-IE2-YFP has been described before [62].

### 2.4. Reporter-Based Viral Replication Assays for Measuring Antiviral Drug Activity and the Parallel Determination of Cell Viability

#### 2.4.1. Antiviral HCMV GFP-Based Reporter Assay

For HCMV AD169-GFP infection, the replication assay was based on a seeding of 225,000 HFFs or 13,500 HFFs in 12 well or 96 well plates, respectively, which were then inoculated with HCMV AD169-GFP [55] at an MOI of 0.25 GFP-FU/mL/7 d for a 90 min period of inoculum–cell adsorption, followed by a medium refreshment including the immediate addition of antiviral compounds. At 7 d p.i., the infected HFFs were either directly fixed with 10% formalin for 20 min at 4 °C (when used for infection in the 96 well format) or lysed and transferred to 96 well plates (when used for infection in the 12 well format) so that the reporter of viral replication could be quantified by GFP fluorometry using a Perkin Elmer Victor X4 Multimode Plate Reader (Perkin Elmer, Waltham, MA, USA).

#### 2.4.2. Further Previously Established Anti-α-, β-, or γ-Herpesviral GFP-Based Reporter Assays

For HSV-1 166v VP22-GFP, HHV-6A U1102-GFP, MCMV C3X-GFP, GPCMV v403-GFP, and EBV Akata-BX1-GFP, the assay conditions were slightly adjusted to those described for HCMV AD169-GFP above and used for automated GFP fluorometric determination as described before in [43,45,57]. To achieve appropriate infection rates and signal intensities in the antiviral assays, the MOIs had to be adjusted individually for the virus infection models (limiting the initial MOIs 0.01–0.0001 at the time point of infection, resulting in a coverage of a maximum of 25% virus-positive cells after the applied viral replication period, when proven to be optimal, to achieve linear concentration-dependent drug sensitivity together with a reliably measurable viral reporter signal).

#### 2.4.3. Newly Established Antiviral Reporter Assays for VZV and KSHV

**Figure 1:**
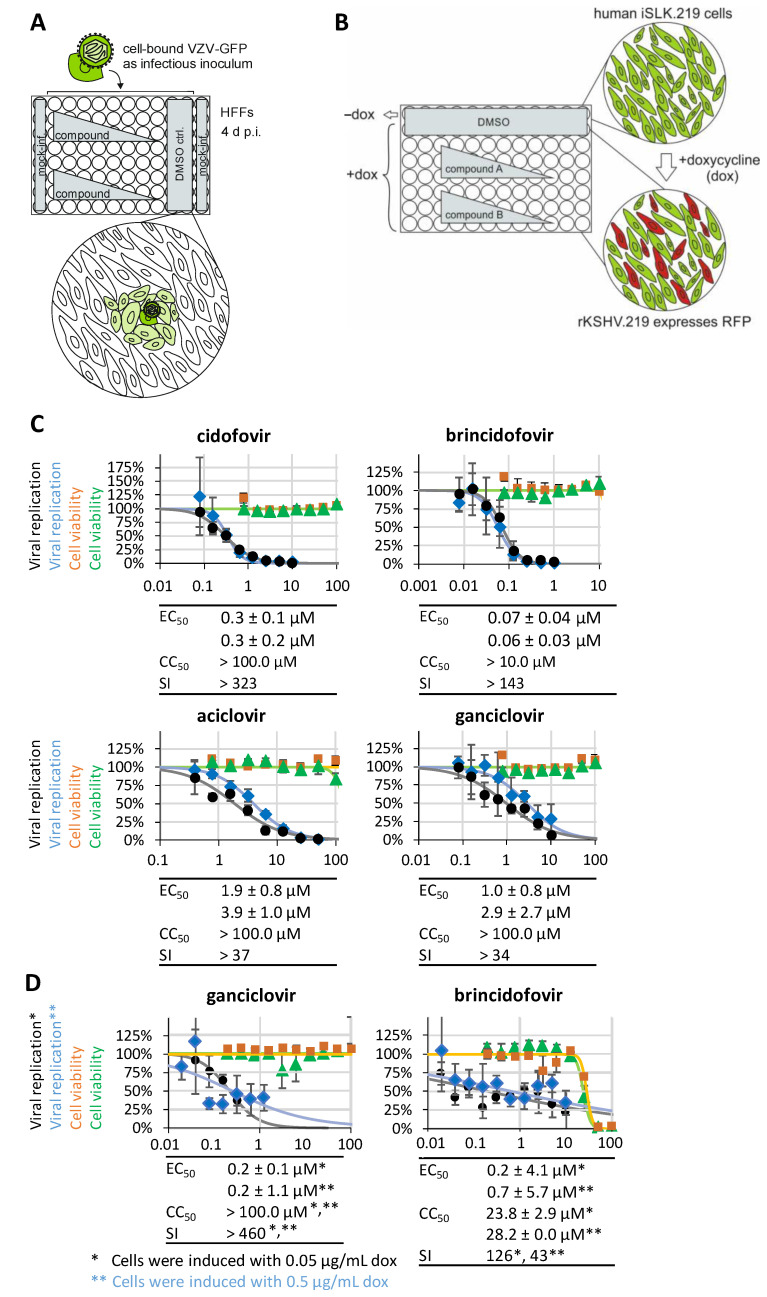
Establishment of new antiviral reporter assays for VZV and KSHV, including their calibration with reference drugs. The virus recombinants VZV Oka-GFP and rKSHV.219-GFP/RFP were used for the infection of HFFs (VZV) or were continuously passaged in iSLK.219 carrier cells (KSHV), respectively, and cell viability was determined by NRA performed with uninfected HFFs or uninduced iSLK.219 cells, respectively. (**A**,**B**) Schematic depiction of the assay systems performed in a 96 well plate format. Both assays can be performed in the 96 well format, which allows working with quite small quantities of material, or alternatively in the 12 well format, which may increase signal intensities. For the VZV-GFP system, virus stock VZV Oka-GFP was applied as a cell-bound infectious inoculum. After a 90 min period of inoculum adsorption to the cell surfaces, antiviral compounds were added to the culture media at serial dilutions (DMSO served as a solvent control for drug treatment of infected cells, while mock-infected cells served as a negative control). For the KSHV-GFP/RFP system, iSLK.219 cell lytic replication of KSHV (RFP reporter signal) was induced by the addition of 0.05 or 0.5 µg/mL doxycycline (dox) to the culture media. Cultivated dox-induced iSLK.219 cells were treated with antiviral compounds added at serial dilutions (DMSO served as a positive infection solvent control, while dox-untreated mock cells served as a negative control). HFFs or iSLK.219 cells were harvested at 4 d p.i. or 1 d after treatment, respectively, fixed by a 10 min incubation with 10% formalin, washed with PBS, and used for quantitation of the GFP or RFP reporter signal by PicoMD counting (ImageXpress^®^ Pico device, Molecular Devices LLC, San Jose, CA, USA), with data evaluation using CellReporterXpress^®^ software (version 2.9.3.1183, Molecular Devices LLC) as described earlier in [63]. (**C**) The antiviral activity of the reference drugs was determined with the VZV-GFP system in the 96 well format. EC_50_ values refer to measurements in quadruplicate, and the mean values ± SD of two experimental replicates are given. (**D**) The antiviral activity of the reference drugs was determined with the KSHV-GFP/RFP system in the 96 well format. EC_50_ values refer to measurements in triplicate, and the mean values ± SD are given for each of the two depicted experimental replicates.

The virus recombinants VZV Oka-GFP and rKSHV.219-GFP/RFP were used for the infection of HFFs (virus stock VZV Oka-GFP applied as a cell-bound inoculum as described before) or were continuously passaged in iSLK.219 carrier cells [61], respectively (Figure 1). The conditions of the GFP/RFP-based assays were adapted to both the 96 well (Figure 1A,B) and 12 well formats, and details including the assessment of antiviral reference drugs are given (Figure 1C,D).

#### 2.4.4. Neutral Red Uptake Assay (NRA)

The cytotoxic level of the compounds was determined by the use of an established quantitation of cell viability (i.e., Neutral Red uptake assay) performed after 3–7 d of compound incubation on uninfected cells (according to the respective duration of the antiviral reporter-based replication assay performed in parallel) as described earlier in [44].

### 2.5. LANCE Ultra CDK-Specific In Vitro Kinase Assay

The inhibitory activity of the QRS compounds toward human CDKs was analyzed by a FRET-based LANCE**^®^** Ultra Kinase Assay (Perkin Elmer, USA) using an ULight™-labeled peptide substrate and an appropriate europium-labeled anti-phospho-antibody. Upon CDK-mediated substrate phosphorylation, the phospho-acceptor peptide site on the substrate was recognized by the europium-labeled anti-phospho antibody. The excitation energy of the europium donor fluorophore at 320 or 340 nm was transferred to the ULight™ acceptor dye on the substrate, resulting in the emission of light at 665 nm, whereby the intensity of the light emission was proportional to the level of peptide phosphorylation. The test compounds were resuspended in DMSO solution to then prepare fourfold serial dilutions for eight doses using the automated liquid handler (POD™ 810, Labcyte, San Jose, CA, USA) so that 80 nL/well of diluted compound solutions were ultimately added into the 384 well plates (Greiner, Kremsmünster, Austria, Cat# 784075). For the CDK-specific assays, 68 nM of ULight-MBP peptide (Perkin Elmer, Cat# TRF0109-M) and 5 µL/well of ATP (Sigma-Aldrich, USA, Cat# A7699) were added to the plates. After 1 min of Eppendorf centrifugation at 1000 rpm, the purified CDK–cyclin complexes were added at the following concentrations: 24 µM for CDK1–cyclin B (Invitrogen, Cat# PR4768C, Carlsbad, CA, USA), 22 µM for CDK2–cyclin A (Invitrogen, Cat# PV6290), 10 µM for CDK5–p25 (Invitrogen, Cat# PR8543B), and 400 µM for CDK7–cyclin H–MAT1 (Invitrogen, Cat# PR6749B). Incubation occurred at 23 °C for 60 min before europium-labeled anti-phospho-myelin basic protein (MBP; Perkin Elmer, Cat# TRF0201-M) and EDTA (Invitrogen, Cat# 15575038) in Lance Detection Buffer (Perkin Elmer, Cat #CR97100) were added to each well. After additional incubation at 23 °C for 60 min, the reaction fluorescence was measured using an EnVision Plate Reader version 2104 (Perkin Elmer, USA; laser as excitation light, with APC 615 nm and europium 665 nm as the first and the second emission filters). Data were analyzed using XL Fit software.

### 2.6. SDS-PAGE and Western Blot Detection of Viral Proteins

SDS-PAGE and standard Western blot analysis, based on equalized amounts of the total cell lysates, were performed as described previously in [64]. The antibodies used for staining were mAb-HA (H9658, Sigma-Aldrich, USA), pAb-Flag (F7425, Sigma-Aldrich), mAb-IE1p72 (P63-27) [65], and mAb-pp28 (both kindly provided by William J. Britt, Dept. Pediatrics Microbiol., Univ. Alabama, Birmingham, AL, USA), mAb-UL44 (kindly provided by Bodo Plachter, Virology, Univ. Mainz, Germany), and mAb-actin (A5441, Sigma-Aldrich).

### 2.7. Assessment of Synergies in Antiviral Drug Combination Treatments

A Bliss independence checkerboard assay and Loewe independence fixed-dose assay were based on the infection of HFFs with HCMV AD169-GFP in a 12 well format and were performed as described before in [45].

## 3. Results

### 3.1. The Discovery of a CDK7-Selective Antiviral Inhibitor with Promising Developmental Features and the Chemical Optimization to Design Covalently Target-Binding Candidate Drugs

In our earlier reports, we described the crucial regulatory roles of cyclin-dependent protein kinases (CDKs), particularly CDK7 [66]. Interestingly, HCMV and other human herpesviruses encode viral CDK orthologs, such as vCDK/pUL97, which possess a multifaceted importance in viral regulation as well as virus–host interaction, and even form complexes with human cyclins and CDKs (particularly a ternary complex of HCMV vCDK/pUL97–cyclin H–CDK7) [46,66]. This sort of orchestrated interplay between cellular and viral kinase regulators immediately implied a variety of inhibitory options that may be utilized for novel antiviral strategies. Thus, the pharmacological inhibition of CDK7 in infected cells may not exclusively block its physiological cellular functionality, but it may specifically produce a block of the virus-supportive CDK7 functions, such as the CDK7-mediated phosphorylation of HCMV regulatory proteins [46], the vCDK–CDK7 complex formation [66], and the trans-stimulation between the two kinases which was identified quite recently [46]. Actually, our investigations provided multiple examples of evidence for the huge and exploitable antiviral potential of CDK inhibitors and vCDK inhibitors, the significant drug synergies demonstrated for CDK + vCDK inhibitor cotreatment, the newly arisen inhibitors blocking vCDK–cyclin complex formation, and likewise the covalently binding warhead compounds, and related inhibitory activities [43,44,45,46,52,67].

**Figure 2:**
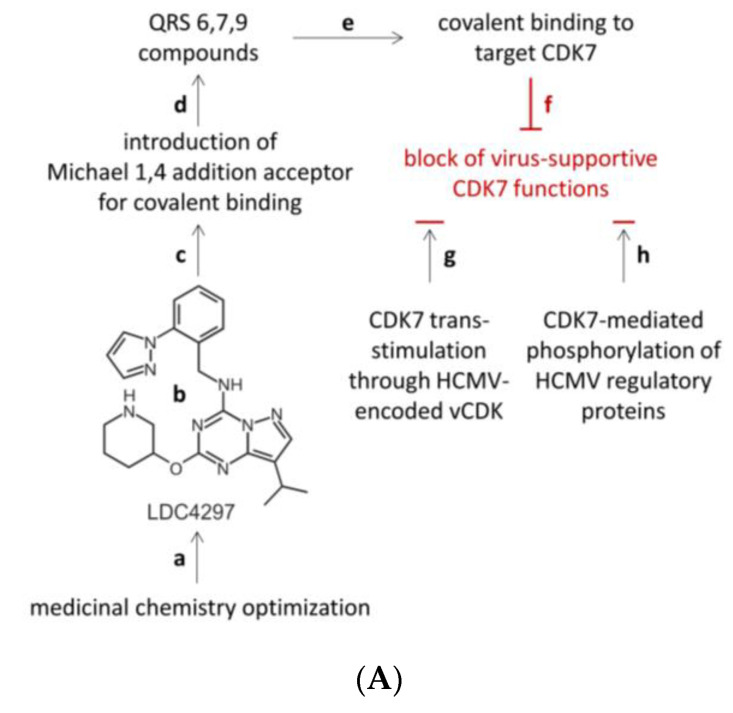

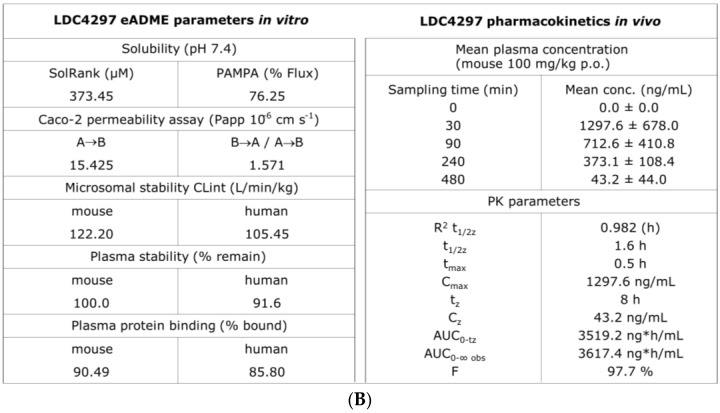
Use of the selective CDK7 inhibitor LDC4297 as the source for generation of novel covalently binding CDK7-directed warheads. (**A**) Chemical linkage of an acceptor for the Michael 1,4 addition reaction was performed to generate QRS compounds 6, 7, and 9. Through the covalent binding of host target CDK7, a block of virus-supportive functions was achieved, and further details on this antiviral strategy were described elsewhere (a,b [43]; c,d, this study, and Yu et al., in preparation; d–f [43,67]; f–h [46,66]). (**B**) Determination of in vitro eADME parameters (early adsorption-distribution-metabilism-excretion) and pharmacokinetics (PK) parameters, including mean plasma concentrations in vivo (male CD-1^®^ mice). For intraperitoneal (i.p.) in vivo applications, the vehicles 30% HP-β-cyclodextrin or 5% Transcutol [52] proved to be most suitable.

Most importantly, we recently developed LDC4297 (Figure 2A), a compound of the chemical class pyrazolotriazines which selectively inhibits CDK7 in vitro in the absence of relevant cytotoxicity [43]. Later, this drug served as the parental compound for the generation of novel covalently binding CDK7-directed warheads (Figure 2A, panels a–h). To reveal LDC4297, a chemical library was designed in silico for the targeting of kinases and was applied for screening against human CDK7–cyclin H–MAT1 by the use of in vitro kinase assays. After medicinal chemistry optimization of the hit series obtained, distinct lead compounds were identified possessing IC_50_ values for CDK7 below 5 nM, among them being the primary hit LDC4297 [43]. In particular, a kinome-wide analysis with 333 individual kinases was performed to assess compound selectivity in vitro. This analysis revealed that LDC4297-sensitive kinases were restricted to members of the CDK family, and among the selected CDK complexes, CDK7–cyclin H–MAT1 showed the lowest residual activity (2%) in the presence of LDC4297. A comprehensive study of the early adsorption-distribution-metabolism-excretion (eADME) and pharmacokinetic properties of this drug in the mouse model revealed the promising qualities of LDC4297 and further investigational CDK7 inhibitors for putative drug development (Figure 2B). All parameters analyzed, including the properties of drug solubility, cell permeability, microsomal and plasma stability, and protein binding, proved to be expedient for proceeding further (Figure 2B, left panel). In addition, the pharmacokinetics in vivo, including measurable mean plasma concentrations of >1200 ng/mL at 30 min, t_½_ of 1.6 h, t_max_ of 0.5 h, and an F value of oral bioavailability of 97.7% (Figure 2B, right panel), also pointed to a highly positive starting situation for producing even further optimized antiviral CDK7 inhibitors.

Building on these findings, we generated pharmaceutically active pyrazolotriazine derivatives as selective inhibitors of CDK7, termed QRS6, QRS7, and QRS9 (Figure 2A). These compounds share with LDC4297 the properties of ATP-competitive inhibition, hinge-binding activity, and structural similarities. However, these novel compounds also show significant structural differences toward LDC4297, such as the distinct covalent binding unit, sugar pocket binder characteristics, and a linker moiety placed between the covalent binding units and hinge regions (structural details confidential at the request of the compound provider; Yu et al., manuscript in preparation). The covalent binding properties of QRS6, QRS7, and QRS9 are mediated through an acceptor of the Michael 1,4 addition reaction (Figure 2A, panels a–d), which typically links the warhead to a cysteine residue of the target protein [68,69,70]. In particular, a linker moiety plays an additionally important role in the high selectivity of QRS6, QRS7, and QRS9 toward CDK7 (Figure 2A, panel e). The goal of these chemical optimizations was the generation of a selective covalent binder that was able to block virus-supportive CDK7 functions (Figure 2A, panels f–h), such as CDK7 trans-stimulation through the HCMV-encoded vCDK/pUL97 [46,66] or the CDK7-mediated phosphorylation of HCMV regulatory proteins [46]. As the drug targeting principle used here, the Michael acceptor mediated the covalent binding of a warhead compound, while the compound scaffold provided main target specificity, which can be individually supported by the coupling of a linker moiety, as designed according to the known target structure.

### 3.2. Antiviral Screening Analysis Performed with the Novel Series of CDK7 Inhibitors

**Figure 3:**
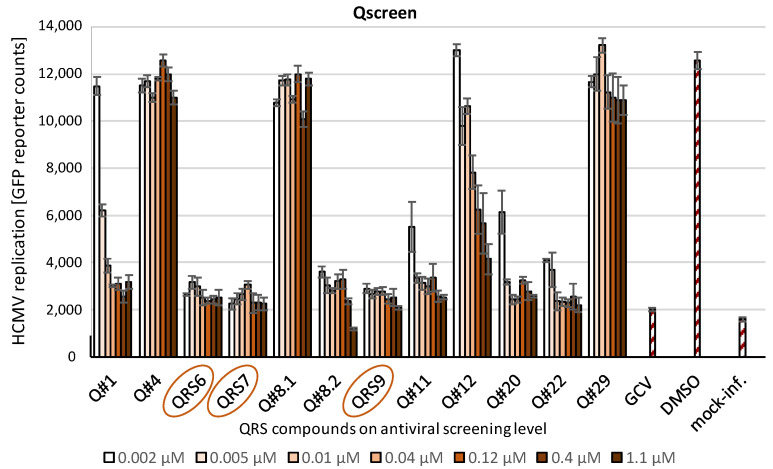
First level of antiviral screening to nominate QRS6, QRS7, and QRS9. Primary human fibroblasts (HFFs) were cultivated in a 12 well format and used for antiviral screening rounds with the HCMV GFP-based replication assay. Infection was performed at an MOI of 0.25 GFP-FU/mL for 7 days (d) before the cells were harvested and the total lysates were used for measurements of automated GFP fluorometry in quadruplicate (infection in duplicates and GFP signal quantitation in duplicates). Note that a representative series of chemically diverse QRS compounds (labeled as Q#) out of a larger number of screening entities is shown, and specifically QRS6, QRS7, and QRS9 of the type of CDK7 covalently binding compounds were identified as the hits exerting a strong anti-HCMV in vitro activity. GCV = ganciclovir reference drug; DMSO = solvent control; mock-inf. = uninfected HFFs as negative control.

In order to address the question about the antiviral potency of novel Qurient compounds (Q#1–Q#29), in particular the in vitro characterized QRS6, QRS7, and QRS9, a round of antiviral screening was performed (termed Qscreen; Figure 3). To this end, an approved experimental reporter system was utilized, as based on the reporter-expressing HCMV AD169-GFP used for the infection of primary human fibroblasts (HFFs), which is applicable in both a 12 well or 96 well format [55,67]. As an important result, several of these covalently CDK7-binding small molecules showed rather pronounced anti-HCMV activity, among these being QRS6, QRS7, and QRS9 (comprising a complete block of viral replication and a reporter signal even at subnanomolar concentrations ≤0.002 µM). Other compounds were either negative or showed intermediate inhibitory phenotypes, which may depend on differences in their pharmacological properties, like cell permeability, intracellular stability, or unwarranted adverse binding effects. None of the screening compounds produced a notable level of cytotoxicity, as monitored by microscopic routine controls and a Neutral Red uptake assay (see the data below). In essence, the three compounds QRS6, QRS7, and QRS9 were identified as experimental hits comprising strong anti-HCMV activity and were thus nominated for further characterization.

In order to verify the target selectivity of the QRS compounds, as previously assured for the parental LDC4297 against human CDK7, the inhibitory activity of the QRS compounds toward human CDKs was analyzed using a FRET-based LANCE^®^ Ultra Kinase Assay (Perkin Elmer) with a ULight™-labeled peptide substrate and europium-labeled anti-phospho-antibody (Table 1; for compound purity, see Figure S1). This assay detects in a quantitative manner the CDK-mediated substrate phosphorylation onto the phospho-acceptor peptide site (as recognized by the europium-labeled anti-phospho antibody, which transfers the europium fluorophore excitation energy toward the acceptor dye on the substrate, thus resulting in a proportional signal of emission light). As an important result, the three hit compounds, QRS6, QRS7, and QRS9, showed pronounced in vitro activity against CDK7 in the low nanomolar range, with IC_50_ values of 0.003 ± 0.001, 0.009 ± 0.003, and 0.01 ± 0.003 µM, respectively (Table 1). While QRS7 and QRS9 additionally proved strict selectivity for CDK7 (and no measurable activity against the other CDKs), QRS6 showed some additional minor reactivity against CDKs 1, 2, and 5 in the micromolar range (approximately 10,000 fold higher than CDK7; Table 1, left panel QRS6). This argued for an achievement of the aspired target selectivity of the covalently binding QRS compounds.

### 3.3. Confirmation Level and Assessment of Anti-HCMV EC_50_, CC_50_, and SI Values

**Figure 4:**
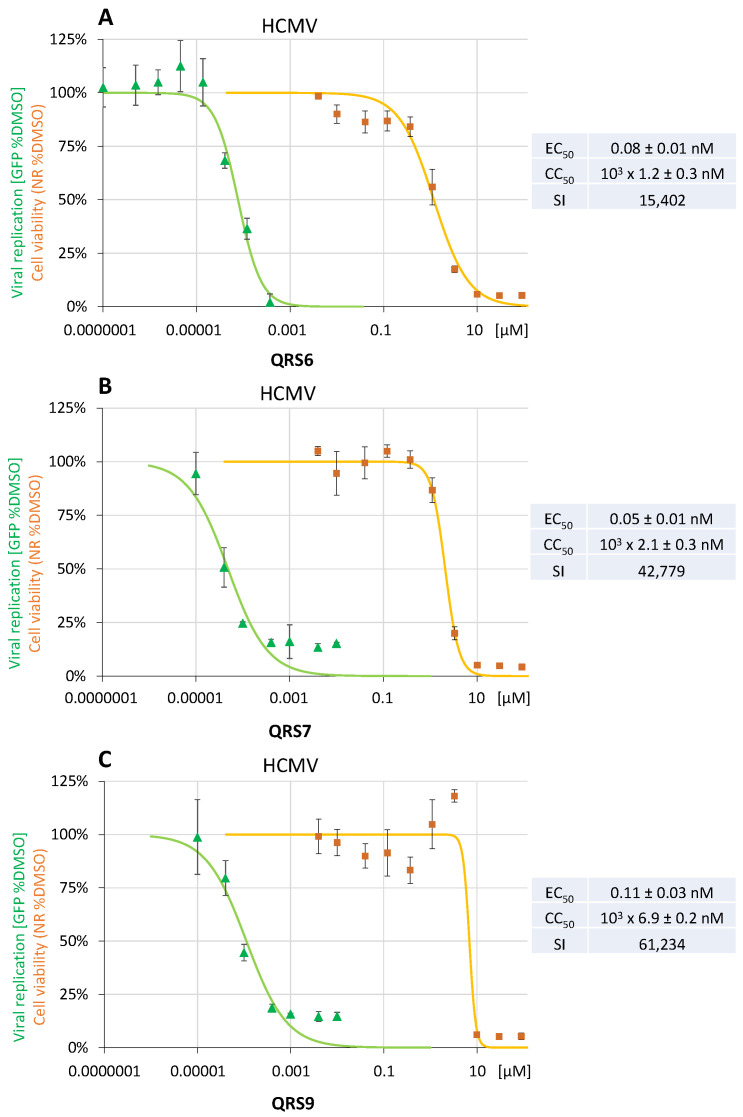
Pronounced antiviral efficacy of the selected CDK7 inhibitor hits QRS6 (**A**), QRS7 (**B**), and QRS9 (**C**) against HCMV in primary human fibroblasts. EC_50_, CC_50_, and SI values were determined by the HCMV GFP-based replication assay, using HFFs in a 12 well format infected at an MOI of 0.25 GFP-FU/mL for 7 d and using measurements of automated GFP fluorometry as performed in quadruplicate (infection in duplicates and GFP signal quantitation in duplicates).

In a second step, the three hit compounds QRS6, QRS7, and QRS9 were specifically assessed for their anti-HCMV activity in quantitative terms. To this end, antiviral activity was assessed using the HCMV GFP-based replication assay in a 12 well setting using HFFs (Figure 4). The EC_50_ values were determined to be 0.08 ± 0.01 nM (panel A), 0.05 ± 0.01 nM (panel B), and 0.11 ± 0.03 nM (panel C) for QRS6, QRS7, and QRS9, respectively, while the cytotoxicity profiles were measured by a standard protocol of the Neutral Red uptake assay using mock-infected HFFs and resulted in mean CC_50_ values of 10^3^ × 1.2 ± 0.3 nM, 10^3^ × 2.1 ± 0.3 nM, and 10^3^ × 6.92 ± 0.2 nM, respectively, thus indicating selectivity indices of >15,000 (SI quotients CC_50_/EC_50_; Figure 4). This finding strengthened the concept of utilizing covalent CDK7 binders for a further investigation of their antiviral properties.

### 3.4. Addressing the Question of Broad-Spectrum Antiherpesviral Activity of QRS Compounds

Based on the finding that the covalent CDK7 inhibitors showed highly pronounced anti-HCMV activity, reaching down to the subnanomolar to picomolar concentration range, we addressed the question of a broader spectrum of antiviral activity, which might include additional herpesviruses. To this end, we performed reporter-based antiviral assays for a selection of human and animal pathogenic α-, β-, and γ-herpesviruses (Table 2). In addition to the human CMV, we also detected strong inhibitory activity at low nanomolar concentrations against murine and guinea pig CMVs, with EC_50_ values between 0.96 ± 0.08 nM and 3.91 ± 0.38 nM, while human herpesvirus 6 type A was insensitive (Table 2; MCMV, GPCMV, and HHV-6). Two α-herpesviruses (i.e., herpes simplex virus and varicella zoster virus) showed low- and intermediate-level drug sensitivity, respectively (Table 2; HSV-1 and VZV). Interestingly, one individual compound, QRS7, exerted rather pronounced activity against VZV at nanomolar concentrations for thus far unexplained reasons. Two γ-herpesviruses (i.e., Epstein-Barr virus and Kaposi sarcoma-associated herpesvirus), likewise showed variable sensitivities with either no detectable or strong-to-intermediate effects (Table 2; EBV and KSHV). These data illustrate, on the one hand, a highly pronounced potency of the QRS compounds against several herpesviruses and, on the other hand, substantial differences in drug sensitivities between individual virus species. The latter point may refer to the individual characteristics of virus–host interaction, as far as CDK7-specific regulatory properties are concerned, which can profoundly differ between the analyzed herpesviruses [71].

### 3.5. Assessment of the Antiviral Mode of Action by an Investigation of HCMV Protein Expression Patterns under Inhibition with QRS Compounds

**Figure 5:**
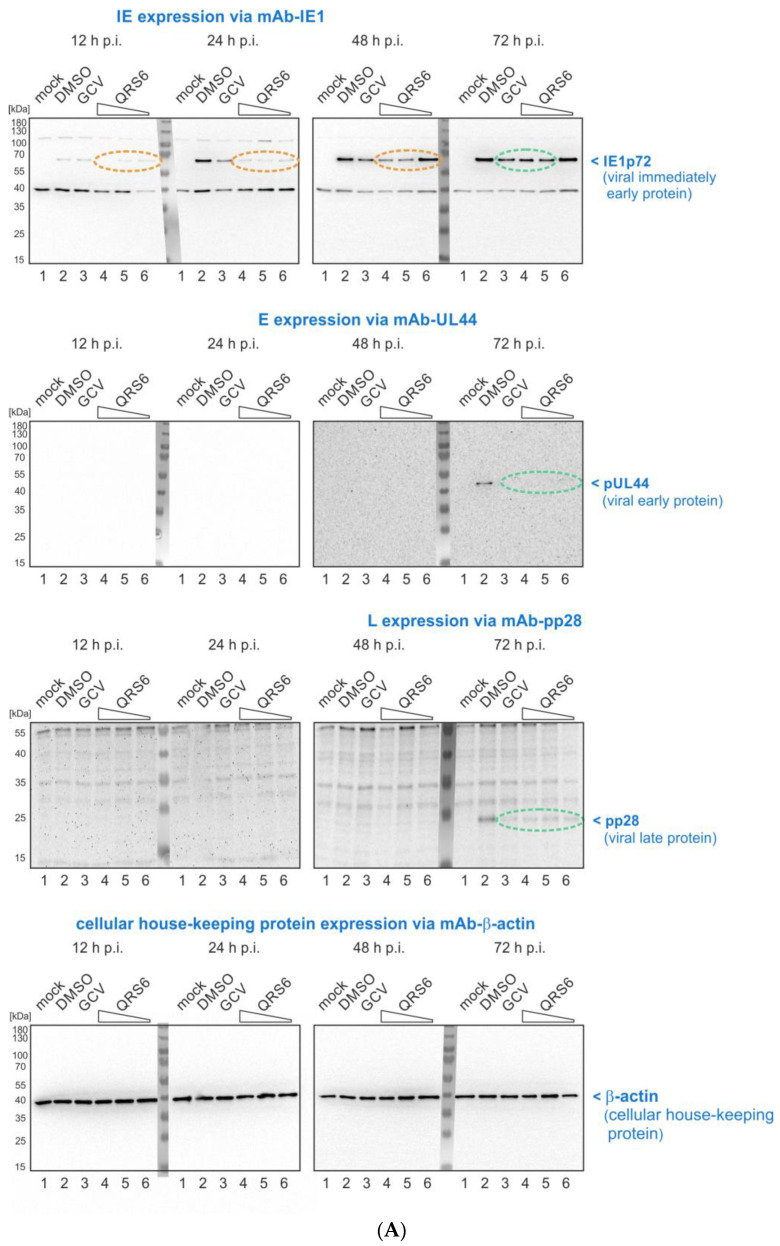

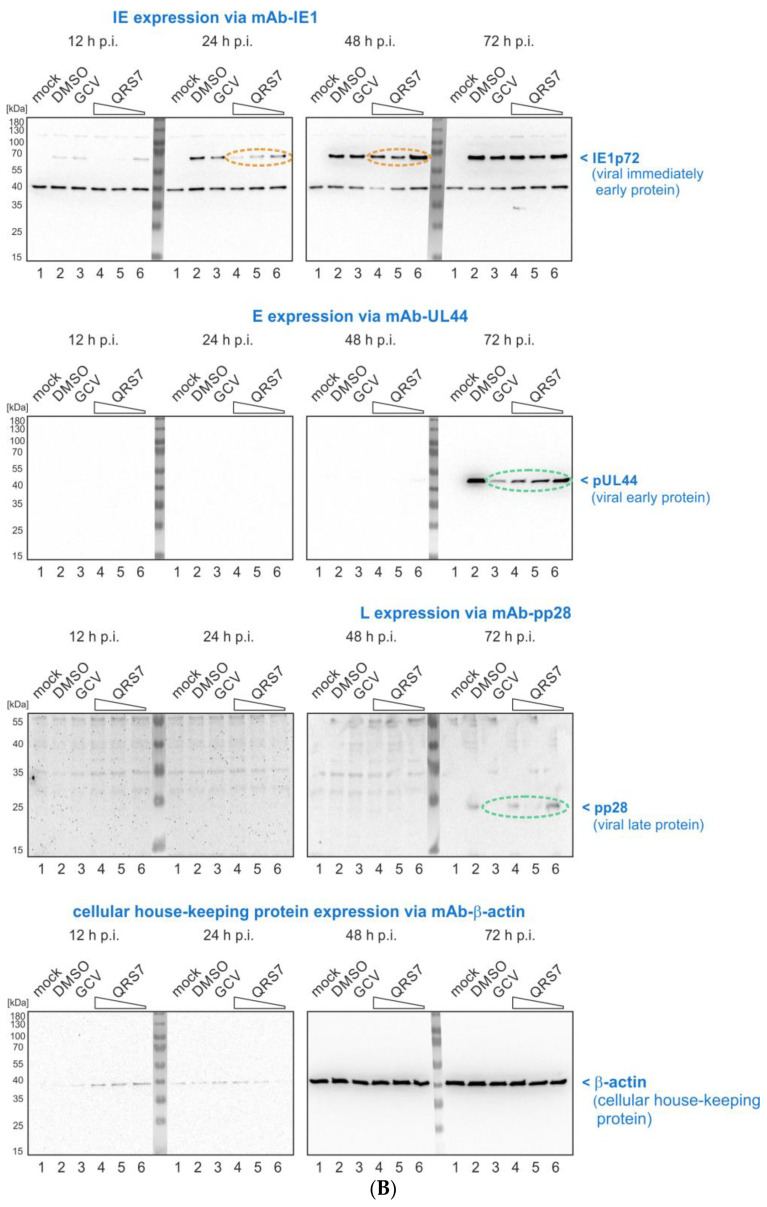

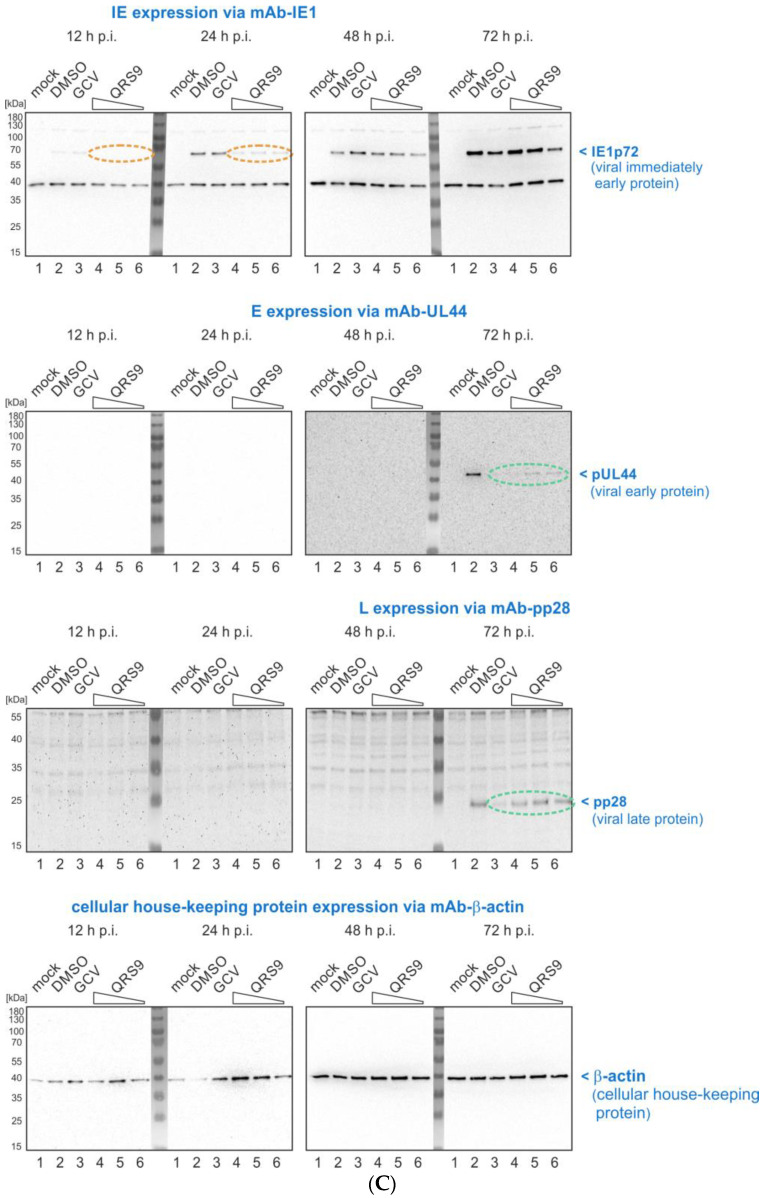
Mode-of-action (MoA) analysis of QRS hits against HCMV. Western blot (Wb) detection of viral IE, E, and L proteins. HFFs were cultivated in 12 well plates and used for infection with HCMV AD169-GFP (approximate MOI of 0.1). At the time points indicated, cells were harvested, used to prepare total lysates, and assayed by standard SDS-PAGE and Wb procedures. For the Wb immunostainings, antibodies against viral proteins of the different stages—immediate early (IE), early (E), and late (L)—of HCMV lytic replication were used (i.e., IE (mAb-IE1/EI1p72), E (mAb-UL44/pUL44), and L (mAb-pp28/pp28)), and mAb-β-actin was used as a cellular housekeeping and loading control protein. (**A**) QRS6. (**B**) QRS7. (**C**) QRS9. Note that those inhibitory effects showing clear differences (orange) in the antiviral MoA compared with the E-specific reference inhibitor ganciclovir (GCV), and those effects showing less pronounced or no difference (green) to GCV were marked by colored ovals. (Note, that primary Wb data are made available upon request).

The putative antiviral mode of action (MoA) of CDK7 inhibitors, especially in the case of HCMV, represents a complex question, since the activity of CDK7 is relevant for various processes important for the efficiency of HCMV replication. This includes virus-induced cell cycle arrest, termed pseudomitosis, transcriptional enhancement in infected cells, and the phosphorylation of viral proteins [46,72,73]. Previous investigations with our parental CDK7 inhibitor, LDC4297, clearly illustrated a rather early block in the viral replication cycle resulting from the inhibition of CDK7 activity (antiviral EC_50_ value of 24.5 ± 1.3 nM) [43]. Analysis of the expression patterns of the viral proteins along the temporal stages of lytic replication identified an inhibitory effect already detectable at the stage of HCMV immediate early (IE) proteins. This IE-directed effect then further translated into the successive stages of viral early (E) and late (L) protein production [43]. A quite similar effect could also be identified in the present study using the covalent CDK7 inhibitors QRS6, QRS7, and QRS9 (Figure 5A–C). For all three compounds, an IE-directed inhibitory mechanism was detectable, as characterized by reduced levels of viral IE1p72 (Figure 5, orange color marks) in the kinetics from 12 h to 72 h post infection (p.i.), later expressed additionally as reduced E (pUL44) and L (pp28) proteins (green color marks). This pattern of an antiviral MoA, as defined here by Wb-based characterization of viral proteins, was distinct from the known MoA of the reference drug ganciclovir (GCV), which is E- or L-specific as directed by the inhibition of viral genome replication. These data indicate a rather rare and unique antiviral MoA of anti-HCMV drugs in the form of an IE-directed inhibition of viral replication.

### 3.6. Synergistic Antiviral Effect Identified by Combination Treatment between QRS6 (CDK7) and Maribavir (vCDK/pUL97)

**Figure 6:**
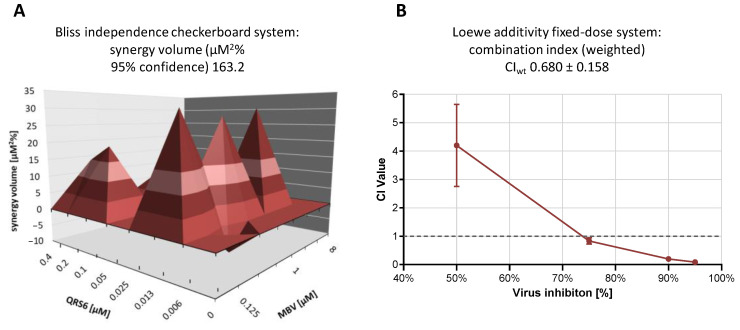
Synergistic anti-HCMV activity demonstrated for the combination treatment QRS6 + MBV. (**A**) Results of the Bliss independence checkerboard assay represented as a synergy plot at 95% confidence interval. Inhibition of HCMV replication by the single compounds at the indicated concentrations was determined, and a theoretical additive inhibition was calculated for each combination of concentrations. Depicted in the planar diagram is the deviation of observed inhibitory effects from this calculated additive value in percentage points, as calculated at a confidence interval of 95% based on three biological replicates. Note that negative synergy volumes (below the 0 µM^2^% plane) indicate antagonistic interaction, and positive volumes (above the 0 µM^2^% plane) indicate synergistic interaction [45]. (**B**) Results of the Loewe additivity fixed-dose assay. Dose–effect curves for each drug were converted to median effect plots before the combination index (CI) values for the drug combinations were extrapolated at 50% (CI_50_), 75% (CI_75_), 90% (CI_90_), and 95% (CI_95_) virus inhibition. Note that the weighted CI of the virus inhibition (CI_wt_) for this combination was 0.680 ± 0.158, which implied a moderate level of synergistic interaction. GFP-based fluorometry was measured in quadruplicate, and mean values ± SD are given.

Recently, the inhibitor of the HCMV vCDK/pUL97, maribavir (MBV), was clinically approved as the first kinase inhibitor for the treatment of a viral infection. Considering the goal to further develop host-directed CDK inhibitors as antiviral drugs, we addressed the question of putative synergistic potential for anti-HCMV combination treatment with QRS + MBV. To this end, two established systems were applied for the statistically robust quantitative assessment of drug interaction: the Bliss independence checkerboard assay and the Loewe additivity fixed-dose assay. The specific parameters and advantages of both assay systems have been reported before [45], and the complementary and affirmative outcome of parallel use has been our approach. In either of the two settings, QRS6 + MBV were applied in serial concentrations of combination treatment, spanning the dosages of previously determined individual dosages of anti-HCMV EC_50_ values. As a clear-cut result, a quantitatively distinct effect of drug synergism was obtained with both approaches (Figure 6). The Bliss checkerboard data indicated a synergy volume of 163.2 with 95% confidence (µM^2^%), and the Loewe fixed-dose data indicated a weighted combination index of 0.733 (CI_wt_). Both results clearly distinguished QRS6 + MBV cotreatment from the areas of additive or antagonistic drug interactions [44,45,74] and provided evidence for a statistically significant example of anti-HCMV drug synergism. This finding may have particular importance for future options of vCDK + CDK inhibitors in antiviral combination treatment.

### 3.7. Lack of Viral Drug Cross-Resistance: Analysis of the Anti-HCMV Efficacy of QRS Compounds against Maribavir- or Ganciclovir-Resistant HCMV Mutants

**Figure 7:**
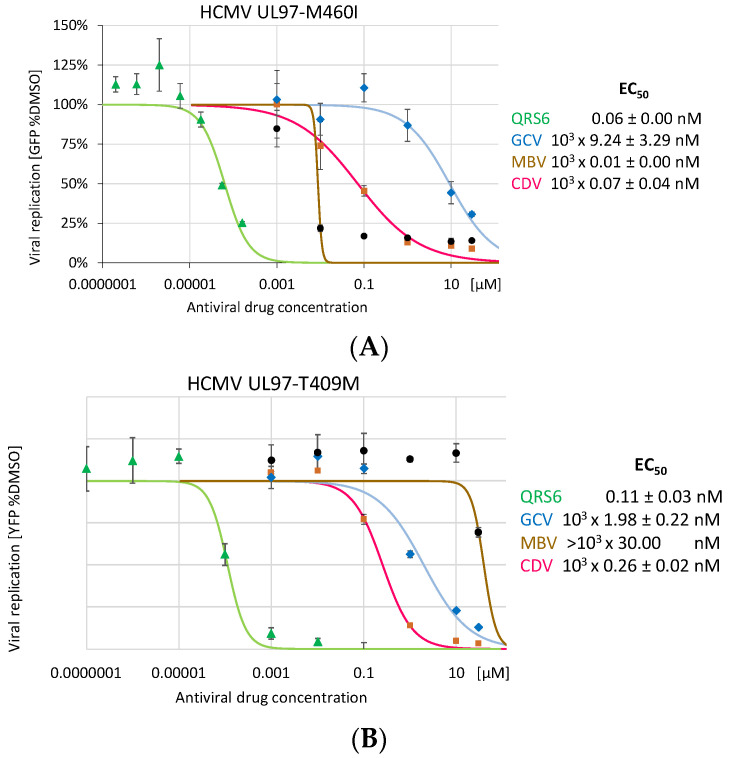

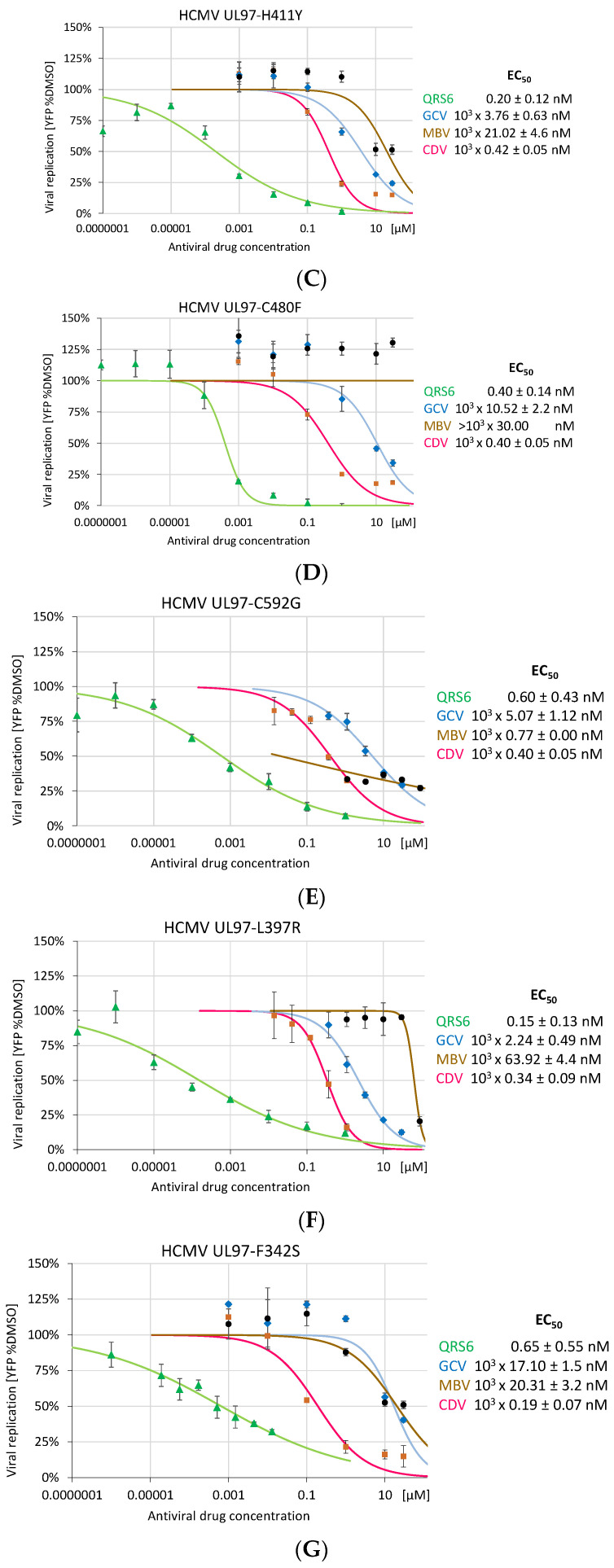

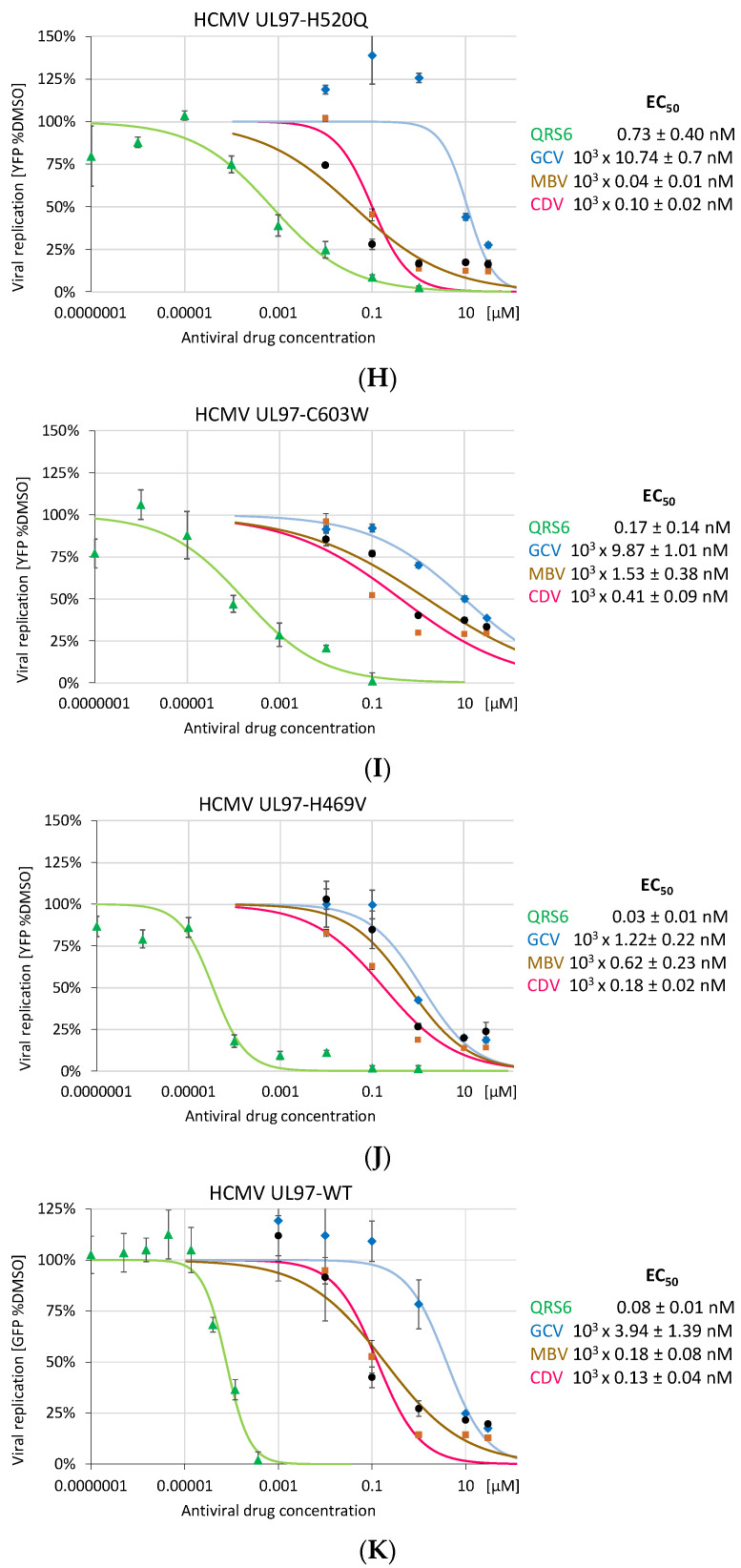
QR6 sensitivity of clinically relevant HCMV mutants carrying UL97 resistance markers against maribavir or ganciclovir. Experimental conditions were identical to those described for Figure 4. HCMV mutants or wild types (WT, AD169-GFP) used were as follows: (**A**) UL97-M460I (based on AD169-GFP); (**B**) UL97-T409M (based on TB40-IE2-YFP, like all following mutants); (**C**) UL97-H411Y; (**D**) UL97-C480F; (**E**) UL97-C592G (**F**) UL97-L397R; (**G**) UL97-F342S; (**H**) UL97-H520Q; (**I**) UL97-C603W; (**J**) UL97-H469V; and (**K**) UL97-WT (wild type).

Due to the fact that the HCMV-encoded protein kinase pUL97 represents a functional and structural ortholog of the host CDKs (vCDK/pUL97), the viral mutants of ORF-UL97 were assessed for sensitivity against CDK7 inhibitors. To this end, a series of known GCV- or MBV-resistant HCMV variants, all carrying drug resistance-conferring mutations in ORF-UL97, was used for addressing the question of the occurrence of any viral cross-resistance phenotype concerning QRS compounds. These HCMVs (i.e., UL97 mutants C592G, H520Q (GCV-res.) L397R (MBV-res.) M460I, T409M, H411Y, C480F, F432S, C603W (GCV- and MBV-double res.), and H469V (not determined yet [75])) were used for comparative analysis in HCMV reporter-based antiviral assays. While the resistance phenotypes of these HCMV mutants could in most cases be confirmed in regard to previous reports [62,75], all viruses showed sensitivity to the reference inhibitor cidofovir (CDV), the activity of which is generally unaffected by ORF-UL97 mutations. Concerning compounds QRS6, QRS7, and QRS9, no signs of drug resistance became evident for these GCV- and MBV-resistant HCMVs (Figure 7). Although the EC_50_ values spanned a range of higher or lower QRS drug sensitivities for these mutants (i.e., low nanomolar to picomolar sensitivities (EC_50_ ranges from 0.03 nM to 0.73 nM, 0.008 to 7.38 nM, and 0.06 to 4.78 nM for QRS6, QRS7, and QRS9, respectively), no indication of cross-resistance was detectable in any of these cases (Figure 7A–J). This conclusion is ultimately illustrated by the summarized data obtained for the anti-HCMV activity of the reference drugs and QRS compounds exerted against this series of viral resistance mutants (Table 3). Thus, the result clearly underlines the unique antiviral mode of action (MoA) of the CDK7 covalently binding QRS drugs, which is independent from viral vCDK/pUL97 kinase activity or ORF-UL97 resistance mutations.

## 4. Discussion

With our approach to broaden the available repertoire of anti-HCMV drugs, mostly composed of direct-acting antivirals (DAAs), we investigated novel host-directed antivirals (HDAs) on the basis of covalently binding CDK7 warheads. The main findings of the present study are as follows: (1) a primary in vitro kinase screening nominated warhead hits with selective binding activity towards CDK7, (2) three of these hits, QRS6, QRS7, and QRS9, exerted antiviral efficacies (HCMV) that reached down to the picomolar levels, (3) the antiviral potency proved to be specifically high against HCMV but (4) also included additional herpesviruses, such as animal CMVs and VZV, (5) the QRS compounds showed a unique mechanistic property in that they inhibited HCMV replication at the immediate early level, (6) in addition, for QRS6 a drug synergy effect was demonstrated for combination treatment with MBV, and finally, (7) all three QRS hits possessed antiviral activity against clinically relevant MBV- or GCV-resistant HCMV mutants without any sign of drug cross-resistance.

As a general remark, the repertoire of drugs that have been developed against the various areas of human disease is immense. Modern approaches include the non-classical mechanistic properties of drugs and non-conventional targeting strategies. As in the focus of our study, it is a matter of fact that covalent drugs have been used for more than 100 years but only gathered larger interest in the last two decades. There are currently over 100 different electrophilic warheads used in covalent ligands, and there are several considerations tailoring their reactivity against the target of interest, which is still a challenging task [70,76,77,78,79,80,81,82]. The exploitation of electrophilic warheads used for protein attacks in chemical biology and medicinal chemistry has been focused upon. The warheads exert specific advantages in covalently binding to targeted residues and in their mechanisms and selectivity, as analyzed by many different datasets. In spite of the numerous electrophilic warheads, only a fraction of them are used in current drug discovery projects. Recent studies identified new tractable residues by applying a wider array of warhead chemistries. However, versatile, selective warheads are not available for all targetable amino acids. Hence, the discovery of new warheads for these residues is needed. Such broadening of the warhead toolbox could result in novel inhibitors, even for challenging targets developing with significant therapeutic potential.

As far as the antiviral mechanism of CDK7 inhibitors is concerned, we have previously shown for the parental LDC4297 that it is unique in a way, as the drug already inhibits cytomegaloviral replication at an extremely early level (i.e., at the stage of IE protein production and activity) [43]. This MoA is distinct from all thus far approved anti-HCMV drugs, which act as DAAs directed to regulatory activities of the viral E, E-L, or L stages of replication. An IE-directed antiviral block may show particularly high efficacy in keeping the viral infectious load at low levels, thus representing a relevant point that needs to be further addressed by in vivo proof-of-concept experimentation [52] and preclinical and clinical investigations. A general conceptional feature of HDAs is their inherent option to achieve a special quality of broad-spectrum antiviral activity. Also in this regard, our previous analysis of CDK7 inhibitors provided information about the high probability of realizing this goal through the CDK7 targeting concept. A relatively broad activity could be demonstrated for LDC4297 against human and animal pathogenic *α*-, *β*-, and *γ*-herpesviruses, as well as some viruses belonging to other families, both in cultured cell infection and animal models [43,44,45,52]. Moreover, drug specificity may be considered as another promising feature of this type of compound, since the investigation of the CDK7 inhibitor LDC4297 in a selectivity panel (i.e., its analysis on a kinome-wide scale spanning 333 human protein kinases) revealed an amazing degree of selectivity for CDKs, especially for CDK7 [43]. This property of mono-selectivity is rather rare among kinase inhibitors, and thus our in-depth analysis of the in vitro characteristics of the parental LDC4297 and novel QRS compounds strongly supported this finding. A recently established highly sensitive and quantitative method of the fluorescence-based in vitro kinase assay (qSox-IVKA) demonstrated the lack of a secondary targeting activity of LDC4297 even against the related HCMV-encoded vCDK/pUL97 [46].

In specific terms, although covalent protein ligands have been avoided for a long period of time (due to potential toxicity issues related to their limited specificity), their advances in the field offer a deeper understanding of binding mechanisms and drug efficacies. As a result, the design of target-specific covalent inhibitors has been intensified, also including efforts to minimize the chance of unwanted side effects. In fact, however, the majority of the drugs approved by the FDA are equipped with warheads exerting promiscuous reactivity [83,84,85,86,87]. But recent reports have clarified that the optimized choice of appropriate noncovalent interactions, which play a crucial role in molecular recognition (as provided by the main scaffold of the warhead compound), combined with the distinctly linked warhead moiety (providing the covalent linkage property), have matched the drug reactivity with specific residues, thus reducing the chance of off-target labeling, even in the case of the promiscuous warheads. Concerning the QRS compounds, we were able to demonstrate that the carefully designed chemical linkage of a covalent binding reactor to a highly CDK7-selective inhibitor as a scaffold resulted in noncytotoxic, selective, and powerful antiviral activity for the candidate compounds. These proved the huge potential for further optimization and drug development toward novel HDAs, probably sharing low-dose efficacy, broad-spectrum potency, and unique mechanistic antiviral properties. However, further studies are required to characterize the bioavailability and in vivo toxicity profiles of these compounds and to identify optimized compounds with improved properties on this basis. Therefore, we are currently focusing on the optimization of these compounds during the preclinical development phase for future clinical studies. This optimization plan includes conducting in-depth pharmacokinetic and pharmacodynamic studies, ensuring safety compliance, and improving the formulation to meet the standards required for clinical application. The goal is to improve the efficacy and safety profile of the QRS compounds, paving the way for progression into the clinical stage. This strategic approach is consistent with the overarching goal of developing next-generation antiviral drugs of the type HDA. From this perspective, the QRS prototype warheads described here may open up thus far unachieved opportunities for a next-generation strategy in antiviral drug targeting.

## Figures and Tables

**Table 1:** LANCE Ultra CDK-specific in vitro kinase assay ^a^.

Human CDKs	QRS6	QRS7	QRS9
**CDK1**	14.4 ± 2.0	>30	>30
**CDK2**	27.4 ± 3.4	>30	>30
**CDK5**	16.5 ± 2.4	>30	>30
**CDK7**	0.003 ± 0.001	0.009 ± 0.003	0.01 ± 0.003

^a^ Data are expressed as IC_50_ values in mean of triplicates ± SD (µM).

**Table 2:** Antiherpesviral EC_50_ values ^a^ of QRS compounds (nM).

Herpesviruses		QRS6	QRS7	QRS9
β	**HCMV**	0.08 ± 0.01	0.05 ± 0.01	0.11 ± 0.03
	**MCMV**	0.96 ± 0.08	3.91 ± 0.12	2.87 ± 0.66
	**GPCMV**	3.91 ± 0.38	nd	nd
	**HHV-6**	>120	>120	>120
α	**HSV-1**	76.9 ± 54.9	83.4 ± 63.2	76.6 ± 34.7
	**VZV**	0.36 ± 0.06	0.003 ± 0.01	1.16 ± 0.25
γ	**EBV**	>1000	>1000	>1000
	**KSHV**	2.65 ± 1.15	8.28 ± 1.07	51.8 ± 13.8

^a^ Cultured cell-based virus replication systems (performed in the 12 well format) with infection of primary human (HFF), murine, guinea pig fibroblasts, or J-Jhan cells. HCMV AD169-GFP, MCMV C3X-GFP, GPCMV v403-GFP, and HHV-6A U1102-GFP for β-herpesviruses (β), HSV-1 166v VP22-GFP and VZV Oka-GFP for infection of HFFs for α-herpesviruses (α), and EBV Akata-BX1-GFP and rKSHV.219-GFP/RFP for infection of Akata B cells or iSLK.219 cells for γ-herpesviruses (γ) (for details, see Section 2).

**Table 3:** QRS compounds: Summary of activity against drug-resistant variants of human cytomegalovirus ^a^.

**HCMV Resistance Genotype**	**UL97-M460I**	**UL97-T409M**	**UL97-H411Y**	**UL97-C480F**	**UL97-C592G**
**Reported phenotype ^b^**	**GVC | MBV | CDV**	**GVC | MBV | CDV**	**GVC | MBV | CDV**	**GVC | MBV | CDV**	**GVC | MBV | CDV**
	R-high | R-low | S	R-low | R-high | S	R-low | R-high | S	R-int | R-high | S	R-int | S | S
**Measured EC_50_ ref. drugs**	**GVC | MBV | CDV**	**GVC | MBV | CDV**	**GVC | MBV | CDV**	**GVC | MBV | CDV**	**GVC | MBV | CDV**
[10^3^ × nM]	9.24 ± 3.3 | 0.01 ± 0.0 | 0.07 ± 0.0	1.98 ± 0.2 | >30.0 ± 0.0 | 0.26 ± 0.0	3.76 ± 0.6 | 21.02 ± 4.6 | 0.42 ± 0.0	10.52 ± 2.2 | >30.0 ± 0.0 | 0.40 ± 0.1	4.67 ± 1.1 | 0.77 ± 0.0 | 0.40 ± 0.1
**Measured EC_50_ QRS**	**QRS6 | QRS7 | QRS9**	**QRS6 | QRS7 | QRS9**	**QRS6 | QRS7 | QRS9**	**QRS6 | QRS7 | QRS9**	**QRS6 | QRS7 | QRS9**
[nM]	0.06 ± 0.0 | 0.13 ± 0.0 | 0.98 ± 0.1	0.11 ± 0.0 | 0.88 ± 0.6| 0.29 ± 0.0	0.20 ± 0.1 | 1.70 ± 0.8| 0.64 ± 0.4	0.40 ± 0.1 | 0.24 ± 0.1| 0.79 ± 0.3	0.60 ± 0.4 | 1.98 ± 1.5| 4.78 ± 3.4
**HCMV resistance genotype**	**UL97-L397R**	**UL97-F342S**	**UL97-H520Q**	**UL97-C603W**	**UL97-H469V**
**Reported phenotype**	**GVC | MBV | CDV**	**GVC | MBV | CDV**	**GVC | MBV | CDV**	**GVC | MBV | CDV**	**GVC | MBV | CDV**
	S | R | S	R-high | R-int | S (for F342Y)	R-high | S | S	R-high | R-int | S	nd | nd | S
**Measured EC_50_ ref. drugs**	**GVC | MBV | CDV**	**GVC | MBV | CDV**	**GVC | MBV | CDV**	**GVC | MBV | CDV**	**GVC | MBV | CDV**
[10^3^ × nM]	2.24 ± 0.5 | 63.92 ± 4.4 | 0.34 ± 0.1	17.10 ± 1.5 | 20.31 ± 3.2 | 0.19 ± 0.1	10.74 ± 0.7 | 0.04 ± 0.0 | 0.10 ± 0.0	9.87 ± 1.0 | 1.53 ± 0.4 | 0.41 ± 0.1	1.22 ± 0.2 | 0.62 ± 0.2 | 0.18 ± 0.0
**Measured EC_50_ QRS**	**[ QRS6 | QRS7 | QRS9**	**QRS6 | QRS7 | QRS9**	**QRS6 | QRS7 | QRS9**	**QRS6 | QRS7 | QRS9**	**QRS6 | QRS7 | QRS9**
[nM]	0.15 ± 0.1 | 0.008 ± 0.0| 0.67 ± 0.4	0.65 ± 0.6 | 1.98 ± 1.5 | 0.58 ± 0.4	0.73 ± 0.4 | 1.97 ± 2.0 | 0.06 ± 0.0	0.17 ± 0.1 | 7.38 ± 3.4 | 0.82 ± 0.1	0.03 ± 0.0 | 0.13 ± 0.0| 0.45 ± 0.1

^a^ Recombinant HCMVs and antiviral test systems were described by our group elsewhere [44,55,57,62]. ^b^ Phenotypes of viral drug resistance were described by other researchers before [75]: R-high, high-level drug resistance; R-int, intermediate-level drug resistance; R-low, low-level drug resistance; S, drug sensitivity; nd, not determined. Colors indicate the phenotypes of drug resistance/sensitivity all analyzed viral mutants (red), as given by previous reports (upper blue) and EC_50_ values measured in this study (lower blue).

## Data Availability

Data is available upon request.

## Supplementary Materials

The following supporting information can be downloaded at https://www.mdpi.com/article/10.3390/pharmaceutics16020158/s1. Figure S1. HPLC spectra used to determine the purity of the analyzed QRS compounds.
