# Supplementary material for: An Antiherpesviral Host-Directed Strategy Based on CDK7 Covalently Binding Drugs: Target-Selective, Picomolar-Dose, Cross-Virus Reactivity
Source: Pharmaceutics. 2024 Jan 23;16(2):158. doi: 10.3390/pharmaceutics16020158 (PMC10892818; doi:10.3390/pharmaceutics16020158)
Supplement: Supplementary file 1 [file pharmaceutics-16-00158-s001.zip › pharmaceutics-2808137-supplementary.pdf]

Supplementary Material

An antiherpesviral host-directed strategy based on CDK7 covalently binding drugs: target-selective, picomolar-dose, cross-virus reactivity

DongHoon Yu[1,*], Sabrina Wagner[2,*], Martin Schütz[2], Yeejin Jeon[1,&], Mooyoung Seo[1,$], Jaeseung Kim[1], Nadine Brückner[2,§], Jintawee Kicuntod[2], Julia Tillmanns[2], Christina Wangen[2], Friedrich Hahn[2], Benedikt B. Kaufer[3], Frank Neipel[2], Jan Eickhoff[4], Bert Klebl[4,5], Kiyean Nam[1], Manfred Marschall[2,#]

[1] Qurient Co. Ltd., C-dong, 242 Pangyo-ro, C801 Bundang-gu, Seongnam-si, Republic of Korea

[2] Institute for Clinical and Molecular Virology, Friedrich-Alexander-Universität Erlangen-Nürnberg (FAU), Schlossgarten 4, 91054 Erlangen, Germany

[3] Institute of Virology, Freie Universität Berlin, Robert-von-Ostertag-Straße 7-13, 14163 Berlin, Germany

[4] Lead Discovery Center GmbH, Otto-Hahn-Straße 15, 44227 Dortmund, Germany

[5] The Norwegian College of Fishery Science UiT, Arctic University of Norway, 9037 Tromsø, Norway

[&,$,§] Present addresses: [&] Kanaph Therapeutics Inc., 112-901, 58, Mokgam-ro, Gwangmyeong-si, Republic of Korea; [$] Mitoimmune Therapeutics, 13th Floor, 108, Bongeunsa-ro, Gangnam-gu, Seoul, Republic of Korea; [§] University Hospital Tübingen, Functional Immunogenomics, Hoppe-Seyler-Straße 3, 72076 Tübingen, Germany.

[*] These authors contributed equally to the study.

[#] Correspondence: manfred.marschall@fau.de, Tel.: +49-9131-8536096

(A) QRS6-specific HPLC report

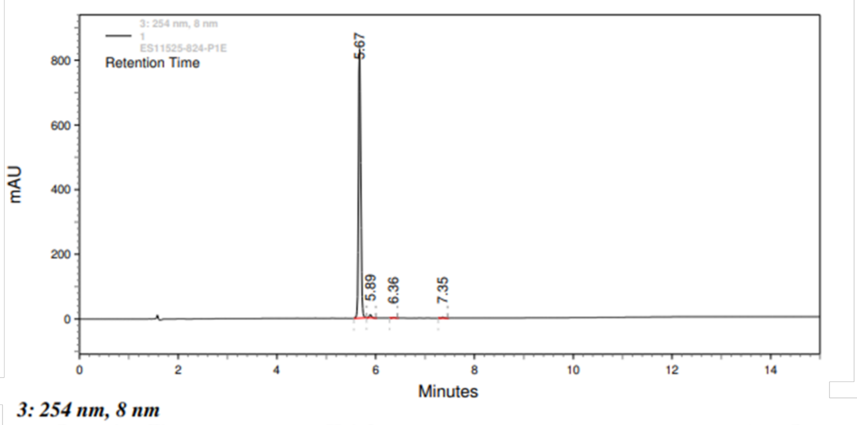

3: 254 nm, 8 nm

Retention Time	*Height*	*Area*	*Area Percent*
5.67	832549	2625098	98.15
5.89	9850	31682	1.18
6.36	2245	8239	0.31
7.35	2953	9650	0.36

(B) QRS7-specific HPLC report

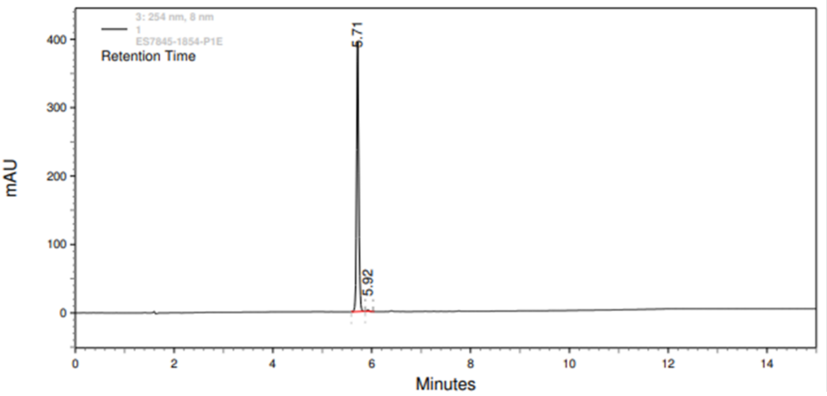

3: 254 nm, 8 nm

Retention Time	*Height*	*Area*	*Area Percent*
5.71	394057	1210799	99.52
5.92	1920	5855	0.48

(C) QRS9-specific HPLC report

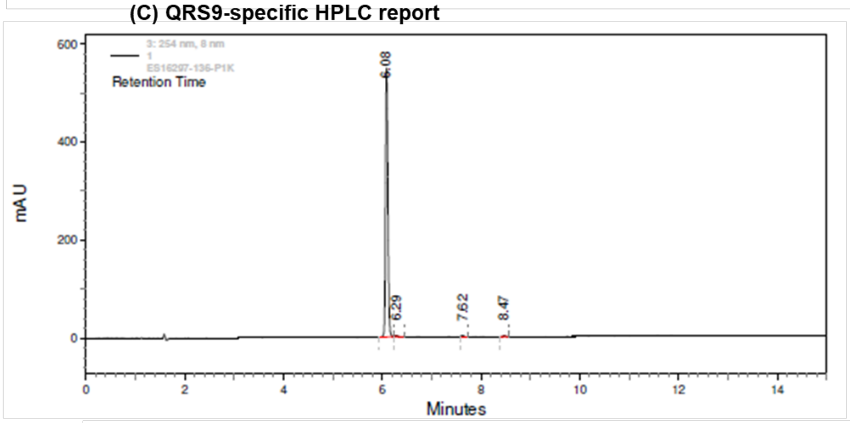

3: 254 nm, 8 nm

Retention Time	*Height*	*Area*	*Area Percent*
6.08	547528	1748824	98.44
6.29	2098	8476	0.48
7.62	3296	9655	0.54
8.47	2296	9655	0.54

Figure S1. HPLC spectra used to determine the purity of the analyzed QRS compounds. Standard high performance liquid chromatography (HPLC) was performed to ensure the required purity of compounds. Spectra and measurement details are given for the compounds as indicated: **(A)** QRS6; **(B)** QRS7; **(C)** QRS9.
